# Enhanced Interferometric Imaging by Rotating Coherent Scattering Microscopy

**DOI:** 10.1021/acsphotonics.5c00123

**Published:** 2025-04-09

**Authors:** Kishwar Iqbal, Jan Christoph Thiele, Emanuel Pfitzner, Philipp Kukura

**Affiliations:** † The Kavli Institute for Nanoscience Discovery, 6396 University of Oxford, Dorothy Crowfoot Hodgkin Building, South Parks Road, Oxford OX1 3QU, U.K.; ‡ Physical and Theoretical Chemistry Laboratory, Department of Chemistry, University of Oxford, South Parks Road, Oxford OX1 3QZ, U.K.

**Keywords:** interference microscopy, interferometric scattering microscopy, rotating coherent scattering, evanescent field, gold nanoparticle, optical contrast

## Abstract

Label-free microscopy on the nanoscale requires high-sensitivity imaging. The challenge of visualizing very small objects, such as nanoparticles, arises from their weak interaction with light. As a result, a combination of high signal-to-noise ratio imaging and background rejection is needed for detection and quantification. Here, we combine concepts from interferometric scattering (iSCAT) microscopy and rotating coherent scattering (ROCS) microscopy to optimize both background rejection and high-sensitivity imaging. Total internal reflection produces a background light intensity more than 2 orders of magnitude stronger than in iSCAT. Despite this, we successfully image 20 nm gold nanoparticles using our combined approach while achieving a signal-to-noise ratio (SNR) comparable to iSCAT at incident power densities as low as 0.04 kW/cm^2^. We experimentally characterize the effect of different incident polarizations and achieve maximal optical contrast using s-polarized illumination. We further demonstrate that ROCS-based illumination at or near total internal reflection yields an approximate 4-fold contrast enhancement and 2-fold background suppression, producing substantially improved SNR compared to iSCAT for the same illumination power entering the microscope objective and integration time. We attribute this to the increased spatial resolution, enhanced incident power density, and rotational averaging of surface-generated speckle. These advantages highlight the potential to achieve and exceed the sensitivity levels attained by related interferometric imaging techniques.

## Graphical abstract

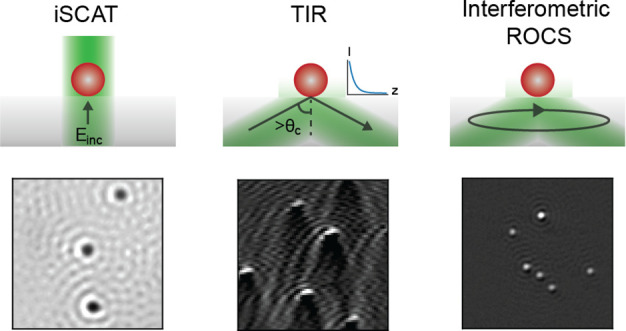

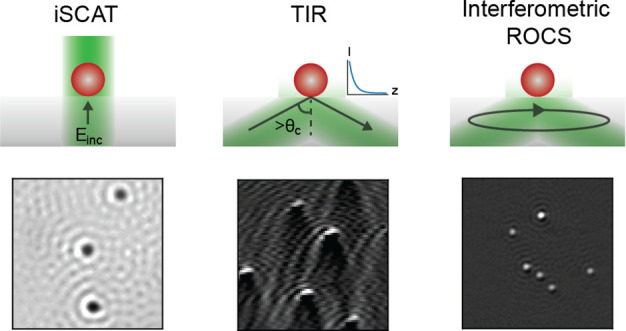

## Introduction

High-sensitivity optical imaging is required for label-free detection and quantification of nanoscale objects, which interact weakly with light. This challenge is central in fields such as biophysics and nanotechnology, where visualizing biomolecules and nanoparticles is necessary to understand structures, interactions, and functions. The core challenge for interferometric imaging is to detect an intensity change as a result of interference caused by a phase shift (Δφ) between a reference and the light that has interacted with the sample.

1 $$I_{d}={|E_{r}+E_{s}|}^{2}={|E_{r}|}^{2}+{|E_{s}|}^{2}+2\left|E_{r}\right|\left|E_{s}\right|\cos\Delta \varphi$$

*I*_d_ represents the intensity at the detector, and *E*_r_ and *E*_s_ denote the reference and scattered fields, respectively. For nano-objects in water bound to a microscope cover slide, the reference field can be provided by the weakly reflective glass–water interface (∼0.5%). In the Rayleigh regime for particle diameters smaller than 50 nm, the polarizability is given by the following:

2 $$\alpha \propto V\frac{n_{p}^{2}-n_{m}^{2}}{n_{p}^{2}+2n_{m}^{2}}$$

where *V* is the particle volume, and *n*_p_ and *n*_m_ are the refractive indices of the particle and the medium, respectively. The scattering cross-section (*σ_s_*) scales with the polarizability and the wavelength of light (λ), following $\sigma_{s}\propto {|\alpha |}^{2}\lambda^{-4}$ . For very weak scatterers, |*E*_s_| ≪ |*E*_r_|, and therefore, |*E*_r_|^2^ = *I*_r_ dominates the image. The relative magnitude of the reference and interferometric terms means that the desired signal appears as a small modulation on top of a large background and is often quantified as the interferometric contrast, given by

3 $$c=\frac{I_{d}-I_{r}}{I_{r}}\approx \frac{2\left|E_{s}\right|\cos\left(\Delta \varphi \right)}{\left|E_{r}\right|}$$

In an ideal experiment, the only limitation to detecting this signal arises from shot-noise-induced fluctuations of the reference intensity, which can be lowered by increasing the number of detected photons. The resulting signal-to-noise ratio (SNR) is given by the following:

4 $$\mathrm{SNR}\propto c\sqrt{N}$$

where *N* is the total number of photons detected, which can be approximated as the number of reference photons per pixel.^1^ Consequently, any object, no matter how low its interferometric contrast, can theoretically be imaged by sufficiently increasing the photon count.

This principle underpins the dramatic improvements in imaging sensitivity afforded by interferometric imaging through phase contrast,^2^ interference reflection,^3^ and interferometric scattering (iSCAT) microscopies,^4^ reaching all the way to label-free imaging and detection of single proteins.^5,6^ Increasing power densities importantly affects not only the detection limit but also the contrast measurement precision, which has enabled the development of mass photometry (MP).^7,8^

An increase in *N* can be achieved by increasing the integration time,^9^ but this approach comes with limitations to measurement concentrations and places strict requirements on instrument stability for ultrasensitive detection and measurement. Similarly, increasing the incident power density is limited by damage to the optics and sample, restricting it to the hundreds of kW/cm^2^ range. Although shorter illumination wavelengths, such as 405 nm, have been used^10^ and enhance scattering signal and thus *c*, further reduction in wavelength is often impractical due to the deterioration of optics, detector efficiency, and sample damage.

As a result, current interferometric measurement approaches at the single-particle level appear to have reached a fundamental ceiling in terms of achievable measurement precision for a given integration time. Further improvements can, in principle, be achieved by alternative enhancement strategies, such as those based on photonic crystals^11^ and plasmons,^12–14^ which are, in principle, attractive because they can produce enormous signal enhancements in the near field. A major limitation in the context of signal quantification, which is central to methods such as MP,^8^ is to ensure that every object produces the same signal, irrespective of its location. This is challenging for enhanced approaches because even nm-scale differences can cause large changes in signal enhancement. While detection sensitivity can be exquisite,^15^ such methods have so far been unsuitable for precise quantification. As a result, the most accurate contrast measurements to date remain those made on plain glass surfaces^8,16^ because they provide a uniform refractive index environment, independent of analyte location.

Another promising approach is evanescent field illumination, widely used in total internal reflection fluorescence (TIR-F) microscopy. It is well-known for its improved imaging capabilities^17–21^ compared to regular epi-illumination as used in reflective interferometric imaging approaches such as interference reflection microscopy (IRM), reflection interference contrast microscopy (RICM), and iSCAT. When incident light strikes the glass–water interface at an angle greater than the critical angle, it undergoes total internal reflection, and the transmitted wave becomes evanescent. The critical angle θ_c_ = arcsin­(*n*_w_/*n*_g_) ≈ 61.7°, where *n*_g_ = 1.51 and *n*_w_ = 1.33 are the respective refractive indices. The resulting exponential decay of the electric field along the *z*-axis confines excitation near the interface and suppresses background noise caused by light penetrating the sample. The enhancement in local illumination intensity can reach a factor of 4–5 depending on the polarization of the incident light.^21^ These characteristics highlight the appeal of an evanescent illumination-based approach.

In label-free microscopy, illumination by total internal reflection has found applications in darkfield imaging^22–27^ where background illumination is suppressed through spatial filtering to isolate scattered photons for detection, *I*_d_ ≈ |*E*_s_|^2^. More generally, such an approach for coherent, oblique illumination—at any angle, not limited to the critical angle—increases resolution through the local interference of adjacent objects in the sample, when sequentially illuminated.^28,29^ However, these images contain coherence artifacts such as edge ringing, speckles, and interference fringes, which have posed a significant problem. Rotationally scanning the beam during a single camera acquisition integrates images from all illumination directions, producing a highly resolved object image and removing artifacts while preserving the benefits of oblique illumination.^29–31^ In addition, this approach restores highly localized, circular point spread functions (PSFs) as opposed to the extended tails associated with oblique illumination. Leveraging these advantages, rotating coherent scattering (ROCS) microscopy has emerged as a high-speed, label-free imaging method demonstrating a spatial resolution of up to 150 nm using a 405 nm laser illumination at 100 Hz frame rates.^10,29,32^ It has been applied to live cell imaging, enabling prolonged observation of dynamic structures without issues such as photobleaching/blinking.^10,33,34^ ROCS is also intrinsically compatible with various imaging modalities including TIR and non-TIR illumination, interferometric/brightfield illumination, and darkfield illumination.^32,35^

Despite these advantages, combining illumination at or near the critical angle with interferometric imaging has been scarce, likely as a result of the up to 200-fold increase in reference light intensity and the resulting dramatic drop in interferometric contrast (see eq 3), making it unsuitable for sensitive imaging of small objects. Ruh et al.^32^ demonstrated the principle of interferometric ROCS at TIR by imaging 200 nm polystyrene beads. They showed that despite a higher detection numerical aperture than in darkfield imaging, the contrast is significantly diminished. Zheng et al.^35^ demonstrated imaging of gold nanoparticles (GNPs) as small as 20 nm with a ROCS illumination-based microscope. They showed interferometric imaging of nanoparticles below an incidence angle of 50°. Beyond this, they transitioned to darkfield ROCS by closing a high numerical aperture diaphragm to cut off the high-intensity reference light to avoid the above-mentioned effect of near total internal reflection. Their approach did not incorporate background subtraction, which is crucial for visualizing and characterizing small GNPs near the critical angle using interferometric imaging.

Here, we present a detailed evaluation of the (dis)­advantages of rotating (near) total internal reflection coupled with interferometric imaging. By characterizing images of multiple 40, 30, and 20 nm gold nanoparticles under varying illumination angles and polarization states, we reveal that the optical contrast does not drop at the rate expected from the increase in reference light. Instead, we show that there is a contrast enhancement due to the improved transfer of high spatial optical frequencies, local field enhancement, and improved background suppression through rotational averaging. These enhancements enable imaging of 20 nm GNPs with improved SNR compared to traditional iSCAT for a given illumination power entering the microscope objective (0.1 mW) and integration time (5 ms). The enhancement in measured contrast coupled with the improved background rejection demonstrated here will serve to push the limits of interferometric imaging, potentially reaching and surpassing the levels of sensitivity recently demonstrated by related approaches.^36,37^

## Experimental Section

### Instrument Setup

We constructed a custom microscope that combines principles from iSCAT and ROCS for imaging (Figure 1a). A 532 nm laser source (Laser Quantum, GEM-532) with a 1 cm coherence length illuminates the sample. A combination of a half-wave plate (HWP) and a polarizing beam splitter (PBS) attenuates the average incident laser power between 0.1 and 6 mW. Dual-axis galvanometer mirrors (ScannerMAX Saturn-5; abbreviated as galvos), which we position conjugate to the sample plane to ensure a fixed illumination area (Figure S1), scan the beam. The combination of a half-wave plate and a vortex half-wave plate (VWP, Thorlabs WPV10L-532) controls the incoming light polarization. A 300 mm field lens focuses the incident light into the back focal plane (BFP) of a 60x, 1.49 NA Olympus objective. A CMOS camera (Blackfly S BFS-U3-17S7M) collects the scattered and reference light, focused by a 750 mm tube lens. A 50:50 beam splitter splits the illumination and imaging pathways. We operate the setup in wide-field (iSCAT), TIR, and rotating TIR (ROCS) modes by changing the command signal to the galvos. Additionally, we constructed a TIR alignment channel to reimage the reflected beam conjugate to the BFP. We measured the scan radius to determine the angle of incidence (AoI) (Figure S2) and maintained optimal alignment for rotational TIR (Figure S3). A *z*-stage (Attocube ECSz3030/Al/NUM/RT) and *y*-stage (Agilis Linear) positioned the sample parallel and perpendicular to the optical axis.

**Figure 1:**
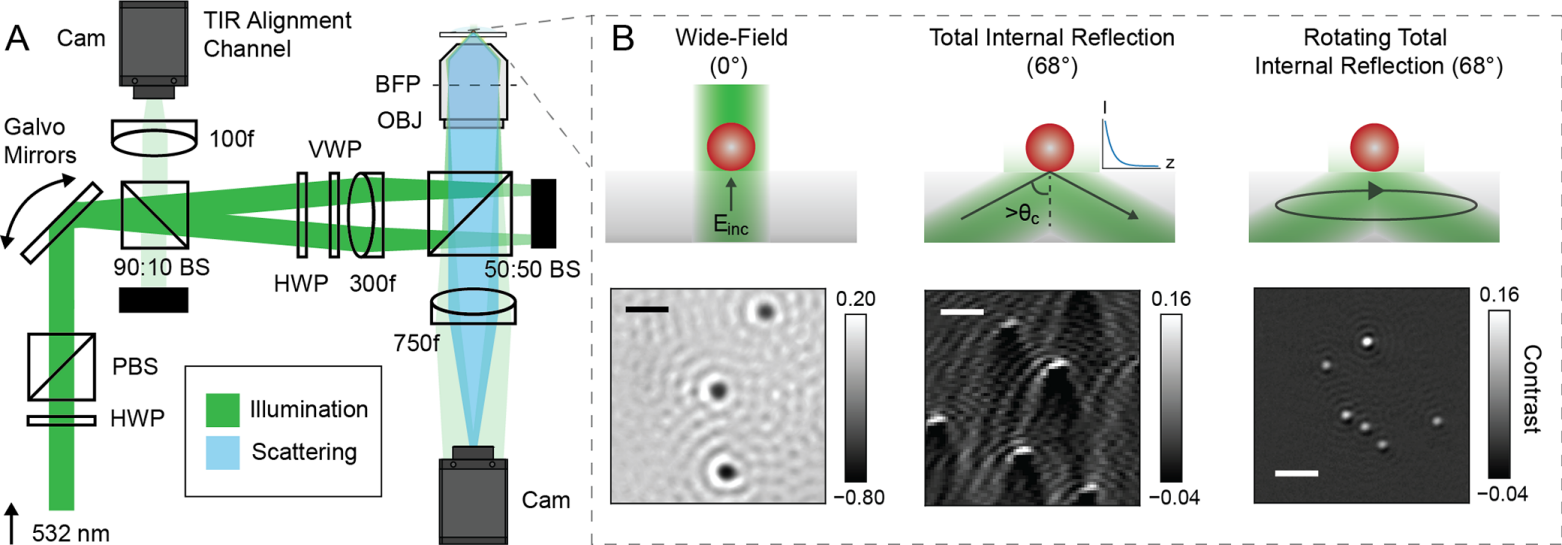
Principle of interferometric rotating coherent scattering. (A) Schematic of interferometric rotating coherent scattering microscopy. (B) Illustration of the various possible imaging modalities for 40 nm gold nanoparticles: wide-field mode (iSCAT)—the sample is illuminated at normal incidence; total internal reflection (TIR) mode—the sample is illuminated beyond the critical angle; and rotating TIR mode (ROCS)—the sample is illuminated beyond the critical angle from all azimuthal directions during a single exposure. Abbreviations: HWP, half-wave plate; PBS, polarizing beam splitter; BS, beam splitter; Cam, camera; VWP, vortex half-wave plate; OBJ, oil immersion objective; BFP, back focal plane. Scale bar: 1 μm.

### Sample Preparation

We functionalized microscope cover slides with biotinylated bovine serum albumin (biotin-BSA) to reduce nonspecific binding. We then introduced streptavidin-coated gold nanoparticles: 20 nm GNPs (C11-20-TS-PBS-50-1, Nanopartz), 30 nm GNPs (EM.STP30, BBI Solutions), and 40 nm GNPs (C11-40-TS-PBS-50-1, Nanopartz). Comprehensive characterization of these nanoparticles has been performed by the respective manufacturers, with relevant details available in the Supporting Information. This functionalization facilitates strong and specific interactions between biotin and streptavidin, reducing aggregation of GNPs and ensuring stable attachment to the surface.

Samples are prepared by following the protocol described by Ortega Arroyo et al.^38^ Briefly, we assemble a flow channel using two clean coverslips (No. 1.5, 24 × 50 mm and 24 × 24 mm). We then incubate the channel with a 0.1 mg/mL solution of biotin-BSA for 5 min, wash it with PBS buffer, and then introduce 0.1 nM streptavidin-functionalized GNPs. After a final wash, we seal the channel with nail polish.

### Data Acquisition and Analysis

During measurement, we circularly scan the beam at 1 kHz synchronized to the 1 ms exposure time of the camera, thereby achieving one full revolution per camera exposure. The cameras are interfaced using LabVIEW, where five frames are binned, resulting in an effective integration time of 5 ms. The setup magnification is chosen to be 250×, yielding an effective pixel size of 36 × 36 nm^2^.

#### Background Removal

A motion-based flat-fielding approach removes static background features (such as nonuniform illumination, unwanted back reflections, optical imperfections, etc.).^38^ To achieve this, we laterally translate the sample to capture 20 different images, from which the median is computed to normalize the raw image, effectively removing stationary features. We performed further processing using the “medfilt2d” function from the SciPy library, with a kernel size of 15, resulting in a background-corrected image (Figure S4).

#### Particle Detection

The combination of a Gaussian filter and a minima/maxima filter smooths the images and identifies particle positions. We threshold the filter output and segment connected components into subregions of interest, creating a binary detection map. We identify candidate particle positions through local minima/maxima within each subregion (Figure S5).

#### Particle Fitting

We extract a 29 × 29 px thumbnail centered around each candidate particle and fit an experimental point spread function to each thumbnail. We then extract the contrast (peak intensity) and the full width at half-maximum (FWHM) (Figure S5).

We repeat this procedure for several particles across multiple fields of view, generating a contrast histogram for the GNP sample. A Gaussian fitted to the contrast distribution extracts the sample contrast. We conducted this process at 8 angles of incidence (7.5 ± 0.2°, 16.2 ± 0.4°, 23.9 ± 0.6°, 35 ± 1°, 52 ± 2°, 61 ± 2°, 68 ± 3°, 71 ± 4°), while rotating the first HWP to attenuate the average laser power to maintain an optimal camera pixel saturation around 80%. Positioning of the second HWP relative to the VWP achieves azimuthal and radial polarizations. Replacing the VWP with a quarter-wave plate (QWP) provides circular polarization (Figure S6). This procedure is performed for each GNP size (40, 30, and 20 nm).

## Results and Discussion

The illumination angle and its azimuthal integration result in significant differences in the acquired interferometric images compared to standard wide-field illumination (Figure 1B). Wide-field illumination yields strong interferometric contrast for 40 nm GNPs in water, with extended interference PSF fringes^39^ and a clearly visible background arising from the nanoscopic roughness of the microscope cover glass.^40^ Moving the incident beam to total internal reflection changes the point spread function to include a comet-like tail. Performing a full 2π azimuthal rotation during a single camera exposure restores a spatially confined, spherically symmetric PSF. This confinement not only improves the measurement precision by minimizing the number of noisy pixels the signal extends over but also facilitates measurement at higher particle density and landing assay concentration, with reduced interfering effects from nearby particles.

The apparent reduction in the size of the PSFs observed across higher incidence angles in Figure 1B is in line with the previously reported resolution enhancement effect of ROCS^32^ (Figure 2). This effective improvement in diffraction-limited resolution contributes to an enhanced interferometric contrast when approaching the critical angle. A Gaussian fit confirms a drop in the full width at half-maximum from 273 to 135 nm when moving from 7.5° to 71.3° (Figure 2A,B). These observed FWHM values are a result of the azimuthally integrated local interferences in ROCS, which also leads to a slight overestimation of distances between neighboring objects.^31^ We computed the image Fourier spectrum for 20 nm GNPs (Figure S7) to accurately determine the spatial resolution^41^ (Figure 2C,D), which remains diffraction-limited. The radial profile of the image spectrum revealed a resolution increase from 307 nm at an incidence angle of 8° to 196 nm at 61°, corresponding to ∼1.5-fold enhancement. For a 2D Gaussian with a preserved integral, a reduction in width by a factor of 1.5 should result in a contrast enhancement of ∼2. This effect also reduces interference from nearby or overlapping particles (Figure S8), which can lead to improved contrast quantification at higher densities.

**Figure 2:**
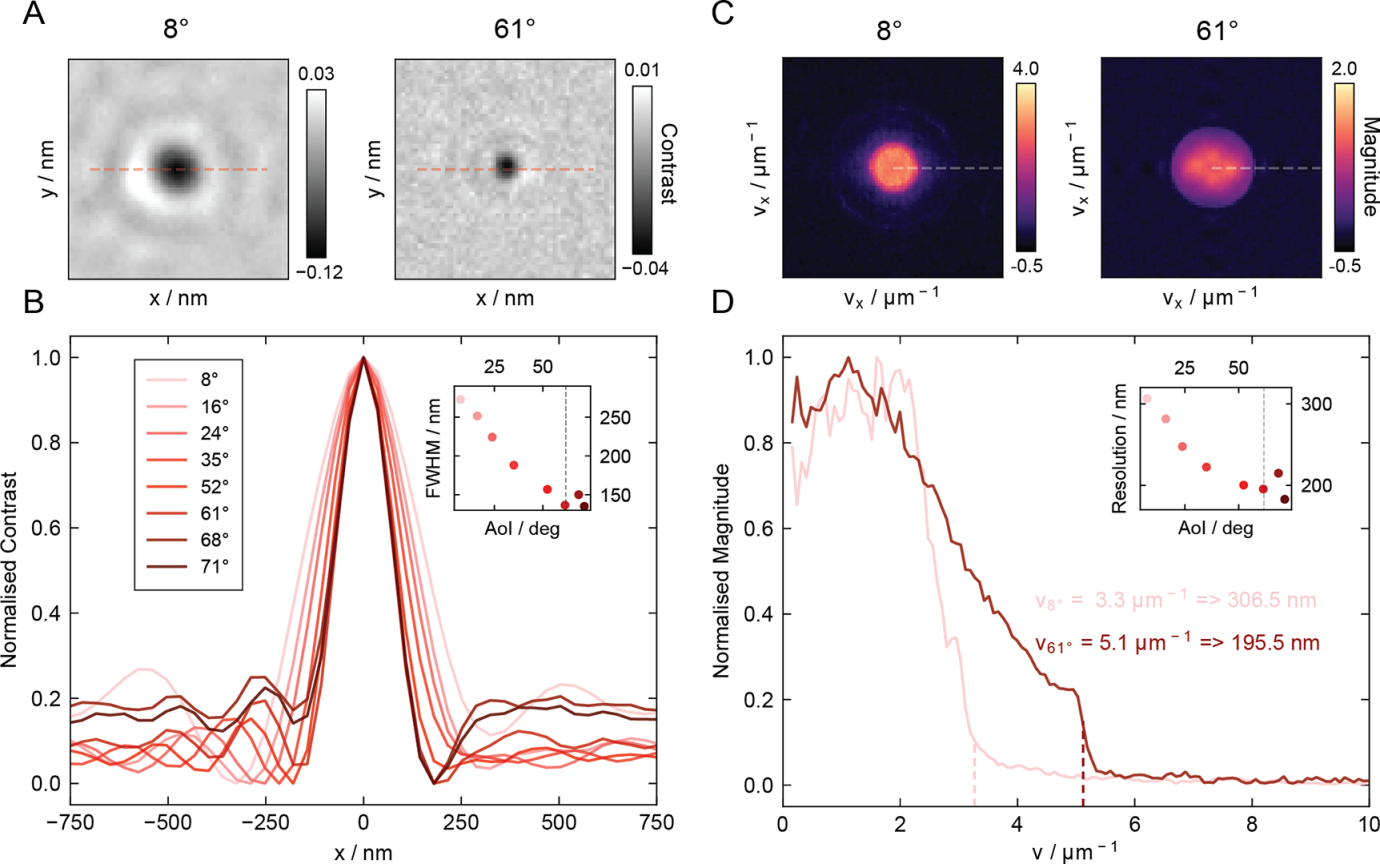
Resolution enhancement for 20 nm gold nanoparticles. (A) Thumbnails displaying a 20 nm GNP illuminated at 8° and 61° angle of incidence. (B) Normalized and averaged 1D linecuts from all 20 nm GNPs observed across varying incidence angles. Inset: FWHM extracted from Gaussian fits to the linecuts as a function of incidence angle. (C) Averaged Fourier spectra of 20 nm GNP images, showing the interference spectrum for azimuthal scanning at 8° and 61°. (D) Averaged radial spectral linecuts demonstrating the diffraction-limited resolution enhancement toward the critical angle. The optical resolution limit was determined at 10× the noise floor. Inset: The observed resolution enhancement across varying incidence angles.

Having confirmed the improved imaging properties of rotating illumination for a sparse coverage of GNPs on glass, we turned to evaluate the impact of the illumination angle and the scaling of the imaging contrast across varying GNP diameters. Offsetting the illumination angle from the center of the objective while azimuthally scanning requires careful management of the incident polarization to ensure maximal optical contrast. Radial polarization results in predominantly p-polarization at the sample and produces distinct donut-shaped point spread functions at high incidence angles (Figure S9), leading to a reduced peak contrast. Circular polarization results in a mixture of s- and p-polarized illumination for all azimuthal angles and thus a mixture of regular and donut-shaped PSFs (Figure S10). Azimuthal polarization results in s-polarization at the sample and produces regular point spread functions with the largest magnitude for a given particle size (Figure 3, in comparison to Figures S9 and S10). We therefore chose this configuration as it provided optimal performance.

**Figure 3:**
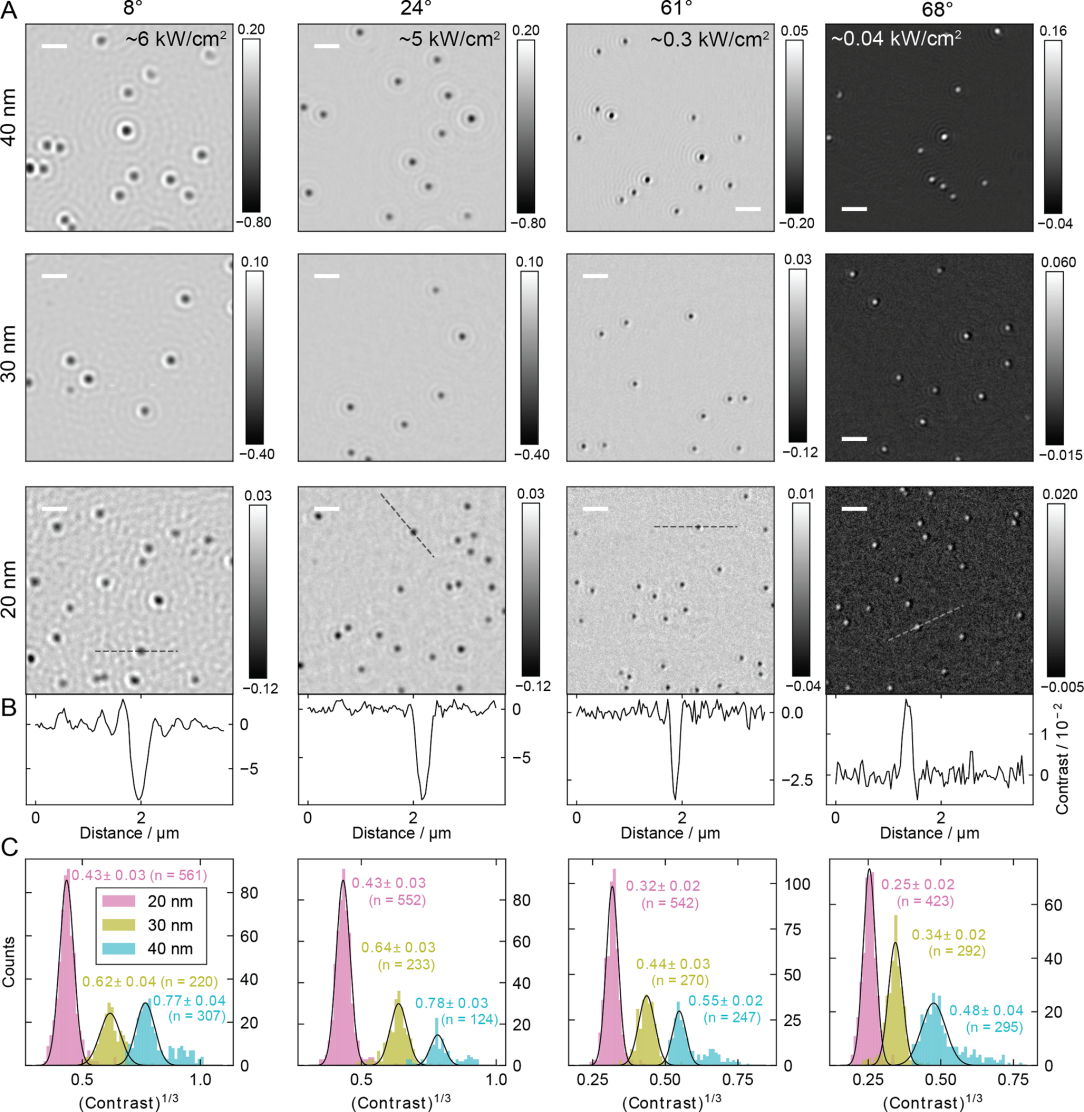
Nanoparticle imaging as a function of incidence angle. (A) Images of gold nanoparticles (40, 30, and 20 nm) measured using interferometric ROCS and characterized across various angles of incidences at 5 ms integration time. Here, s-polarized light illuminated the sample, provided from azimuthally polarized light entering the objective. The incident power density is varied between 6 and 0.04 kW/cm^2^ to maintain constant camera counts throughout the measurements. (B) Representative linecuts for 20 nm particles. (C) Contrast histograms for each nanoparticle sample across various incidence angles, generated from several images. Color bars: Contrast. Scale bars: 1 μm.

Compared to traditional wide-field iSCAT at normal incidence—captured over a 14 μm × 14 μm field of view (cropped image shown in Figure 1B) illuminated with a Gaussian beam of approximately 10.5 μm FWHM—ROCS illumination effectively eliminates unwanted back reflections from the objective and minimizes image speckles (Figure 3A,B). The Z-position of the coverslip affects particle contrast (Figure S11), with comparable contrast levels observed at both the largest constructive and destructive interference peaks. When translating the sample through the focus along the optical axis, we found maximal contrast for the destructive interference peak, resulting in dark particles^42^ below the critical angle. Interestingly, above the critical angle, we observed maximal contrast at the constructive interference peak, resulting in bright particles. We believe this behavior is associated with the phase shifts experienced by reflected light that accompany total internal reflection.^21^ In Figure 3C, we evaluate the contrast for GNP samples of different diameters at varying incidence angles, which is in good agreement with the diameter-cubed contrast scaling predicted by Rayleigh scattering (Figure S12). The 20 and 30 nm GNP samples exhibit contrast distributions that are well-described by a normal distribution. However, the 40 nm sample shows a high-contrast tail, likely resulting from GNP aggregation. While this may introduce a bias in the extracted mean contrast, its impact on the relative contrast changes across incidence angles remains minimal.

For 20 nm GNPs, we found a contrast of 8 ± 2% at near-normal incidence (8°). This value is comparable to traditional iSCAT with acousto-optic deflector (AOD) scanning.^38^ While we find that the contrast of the GNPs decreases with increasing incidence angle, it does not drop at the rate expected from the increase in reference light intensity, which changes by a factor of 200 between epi- and TIR-illumination (Figure 3C). In addition, in traditional iSCAT, 20 nm GNPs exhibit a signal-to-background ratio (SBR) of approximately 10,^25,38,43^ limited by the underlying glass roughness. Close inspection of our images and the accompanying linecuts reveals a consistently high SBR, even as the incident power decreases rapidly to 0.04 kW/cm^2^ at high angles. Notably, significant background suppression is already achieved at illumination angles greater than 15°.

We further investigate this effect in Figure 4, where linecuts from measurements at each incidence angle are superimposed, illustrating the suppression of glass roughness features (Figure 4A). To isolate the image background and remove the influence of shot noise, we increased the integration time to ∼1 s (Figure 4B). The SBR was calculated by comparing the standard deviation of the background signal to the average measured signal for 20 nm GNPs (Figure S13) and plotted as a function of incidence angle (Figure 4C). This demonstrates an ∼2-fold increase in the SBR between 16° and 61° compared to near-iSCAT at 8°. Our analysis suggests that the improvement ceases beyond the critical angle. The reduced PSF, with diminished fringes extending outward, also contributes to the observed background suppression. While these fringes do not vanish entirely, we find that even for a relatively dense 20 nm gold nanoparticle sample, the background suppression remains comparable to that of a sample with little to no particles (Figure S14).

**Figure 4:**
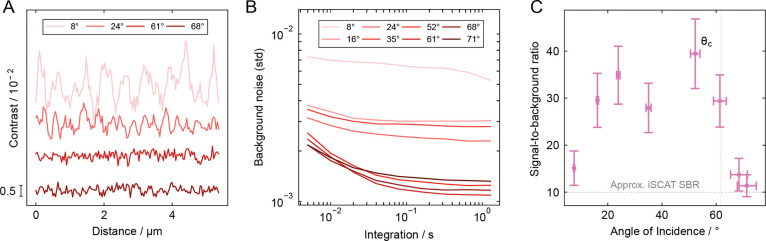
Signal-to-background ratio analysis for 20 nm gold nanoparticles. (A) Background linecuts extracted from images of 20 nm gold nanoparticles acquired at various incidence angles with ∼1 s integration time. Plotted with an applied vertical offset for clarity. (B) Average background noise, quantified as the standard deviation of background contrast signal, plotted as a function of integration time. (C) Signal-to-background ratio at ∼1 s integration time, calculated using the average contrast signal measured for 20 nm gold nanoparticles.

Returning to the observed effect on the interferometric contrast, the increase in reflectivity with incidence angle (Figure 5A, top) is accompanied by a ∼2-fold enhancement in the illumination field amplitude at the critical angle (Figure 5A, bottom).^44^ When coupled with the additional factor of 2 in contrast gained from a reduced PSF, this effect explains why our observed contrast (Figure 5B) does not decrease by the factor of ∼16 expected from the increased field reflection coefficient (1 at 68° compared to 0.06 at 8°). We calculated the reflectivity-independent enhancement from the ratio of the measured contrast (*c*_θ_) and relative reference field ( $E_{r}^{\theta }/E_{0}$ ) for a specific incidence angle (θ), compared to those at low incidence in a traditional iSCAT regime: $c_{\theta }E_{r}^{\theta }/c_{\mathrm{iSCAT}}E_{r}^{\mathrm{iSCAT}}$ , where *E*_0_ is the incident field. The averaged values across measurements of 40, 30, and 20 nm GNP samples exhibit an enhancement factor of 3 ± 0.2 at 68° in total internal reflection (Figure 5C). The enhancement factor increases to 4 ± 2 very close to the critical angle, where local illumination enhancement is the highest, albeit with substantial uncertainty arising from the strong influence of errors in the incidence angle.

**Figure 5:**
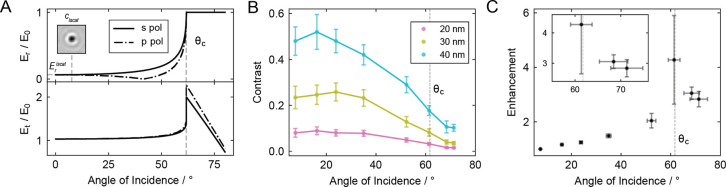
Contrast enhancement for rotating interferometric scattering. (A) Top: relative reflected field by the glass–water interface for s- and p-polarized light. The critical angle is denoted by θ_c_. The reflected field for traditional iSCAT at near-normal incidence ( $E_{r}^{\mathrm{iSCAT}}$ ) is shown, with an inset of a 40 nm gold nanoparticle recorded in this regime. Bottom: relative local field by a glass–water interface (evanescent beyond the critical angle). (B) Contrast values measured for 40, 30, and 20 nm gold nanoparticles as a function of the angle of incidence. The error bars represent the width of the Gaussian fitted to the sampled distribution. (C) Reflectivity-independent contrast enhancement factor at increasing incidence angles compared to normal incidence. Values are averaged across the three GNP samples, with error bars representing the combined error from the standard error of the mean and the uncertainty associated with the measured incidence angle.

To quantify the SNR, we used 5 ms integration images from Figure 3, where shot noise dominates the background-subtracted images at higher incidence angles and lower power densities. We estimate the noise from the standard deviation of a 1D linecut of the image background and compare it to the averaged particle signal (Figure S13). For 20 nm GNPs (Figure 6A, solid line), we find that high SNR is maintained with increasing incidence angles, with only a slight drop-off by a factor of 2, from 17 ± 4 (8°) to 8 ± 2 (TIR) despite a drop in incident power density by 2 orders of magnitude. This highlights the importance of the contrast enhancement introduced by rotating total internal reflection illumination. Without this enhancement, the SNR values for the measured 20 nm GNPs would drop below the generally detectable threshold of 3 (Figure 6A, dashed line), resulting in much lower visibility.

**Figure 6:**
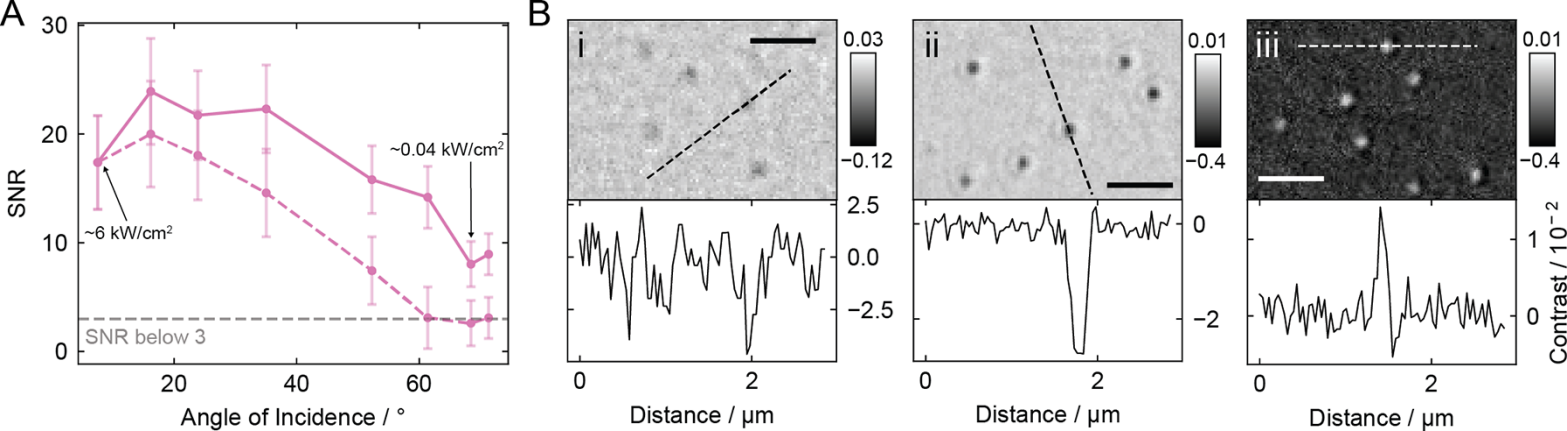
SNR and enhancement for a given power and integration time. (A) SNR for 20 nm GNPs across various incidence angles (solid line) at 5 ms integration and constant camera counts (varying power density), compared to the corresponding SNR without the calculated contrast enhancement (dashed line). (B) Images of 20 nm GNPs with ∼0.1 mW illumination power entering the microscope objective at 5 ms integration. At an incidence angle of 8° (i, approximate SNR ≤ 3), 61° and 68° with measured enhancement (ii and iii, approximate SNR ∼ 10). Color bars: Contrast. Scale bars: 1 μm.

Specifically, we demonstrate that rotating illumination at or near total internal reflection substantially enhances the SNR (by a factor of at least 3) for a given illumination power entering the objective and integration time (∼0.1 mW, 5 ms), leading to improved detection and precise measurement of 20 nm GNPs. This is evident when we compare the images acquired at 61° or 68° to traditional iSCAT, as shown in Figure 6B. Both 61° (near TIR) and 68° (at TIR) incidence angles offer advantages, though the optimal choice remains within the error of our measurement. Both angles produce spherical and confined PSFs, with speckle and artifact suppression, as well as improved resolution and contrast. At 61°, our analysis demonstrates superior background suppression. As shown in Figure 5, the largest illumination field enhancement, and thus the greatest contrast boost, occurs very close to the critical angle. However, the steep gradient of the reference field near TIR can lead to increased uncertainty, instability, and challenges in alignment, whereas operating at 68° provides a more stable profile of the reference light. In conclusion, we show that rotating illumination at or near TIR provides significant advantages over traditional wide-field iSCAT, enhancing contrast, suppressing background, and achieving high SNR/SBR imaging, whether at low power or with the same power and integration time (avoiding camera saturation).

We have demonstrated the combination of iSCAT with (TIR-)­ROCS microscopy to image 40, 30, and 20 nm GNPs. Despite the increase in reflectivity when approaching the critical angle, which required significantly attenuated average power densities from 6 to 0.04 kW/cm^2^ to avoid camera saturation, we successfully imaged 20 nm GNPs with comparable SNR to traditional iSCAT. Notably, we show substantially improved SNR for a given illumination power entering the objective and integration time. We attribute this observation to the roughly 4-fold contrast enhancement, resulting from reduced PSF width and enhanced incident power density coupled with increased background suppression provided by rotational averaging. Our approach facilitates straightforward multicolor integration, phase imaging capability,^45^ the ability to probe sample orientation by introducing a z-component of polarization as widely demonstrated in TIR-F microscopy,^21^ and increased concentration measurements for landing assays. While the contrast for 20 nm GNPs ultimately decreased from 8 ± 2% to 1.6 ± 0.4% in TIR mode, there is scope to implement amplitude control, as previously demonstrated,^7^ to achieve superior sensitivity, even at the single-molecule level. These advances, which enhance SNR and improve SBR, while bypassing practical constraints on power density and integration time, have immediate potential to improve single-molecule mass measurements and redefine the limits of sensitivity in interferometric imaging.

## Supplementary Material



## Notes

Additional details on the methods, results, and analyses performed in this study are presented. It includes calculations of the angle of incidence and alignment for total internal reflection mode, as well as descriptions of the background subtraction procedures, particle detection methods, and fitting techniques. Experimental results are presented for gold nanoparticles under radial and circular incident polarization. Additionally, calculations of signal-to-background and signal-to-noise ratios are provided to support the findings (PDF)
