# Supplementary material for: Enhanced Interferometric Imaging by Rotating Coherent Scattering Microscopy
Source: ACS Photonics. 2025 Apr 9;12(5):2647–55. doi: 10.1021/acsphotonics.5c00123 (PMC12100722; doi:10.1021/acsphotonics.5c00123)
Supplement: Supplementary file 1 [file ph5c00123_si_001.pdf]

# Enhanced interferometric imaging by rotating coherent scattering microscopy

*Kishwar Iqbal[1,2], Jan Christoph Thiele[1,2], Emanuel Pfitzner[1,2], Philipp Kukura[1,2]*

1) The Kavli Institute for Nanoscience Discovery, University of Oxford, Dorothy Crowfoot Hodgkin Building, South Parks Road, Oxford OX1 3QU, UK

2) Physical and Theoretical Chemistry Laboratory, Department of Chemistry, University of Oxford, South Parks Road, Oxford OX1 3QZ, UK

## Supporting Information

The Supporting Information provides additional details on the methods, results, and analyses performed in this study. It includes calculations of the angle of incidence and alignment for total internal reflection mode, as well as descriptions of the background subtraction procedures, particle detection methods, and fitting techniques. Experimental results are presented for gold nanoparticles under radial and circular incident polarization. Additionally, calculations of signal-to-background and signal-to-noise ratios are provided to support the findings.

### 1. Gold nanoparticle characterisation

<u>40 nm gold nanoparticles</u>
Streptavidin-functionalized spherical gold nanoparticles (C11-40-TS-PBS-50-1) were sourced from Nanopartz. According to the provided certificate of analysis, these nanoparticles have a measured diameter of 40 nm with a size polydispersity index (PDI) of 9%, as determined by dynamic light scattering (DLS) and transmission electron microscopy (TEM).

<u>30 nm gold nanoparticles</u>
Streptavidin-functionalized spherical gold nanoparticles (EM.STP30) were sourced from BBI solutions. The mean diameter of the nanoparticles is 30.0 nm, with a coefficient of variation of 6.7%, as reported in the manufacturer's quality control data. Additionally, 98% of the particles are confirmed to be single nanoparticles, ensuring minimal aggregation.

<u>20 nm gold nanoparticles</u>
Streptavidin-functionalized spherical gold nanoparticles (C11-20-TS-PBS-50-1) were sourced from Nanopartz. According to the provided certificate of analysis, these nanoparticles have a measured diameter of 20 nm with a size polydispersity index (PDI) of 10%, as determined by dynamic light scattering (DLS) and transmission electron microscopy (TEM).

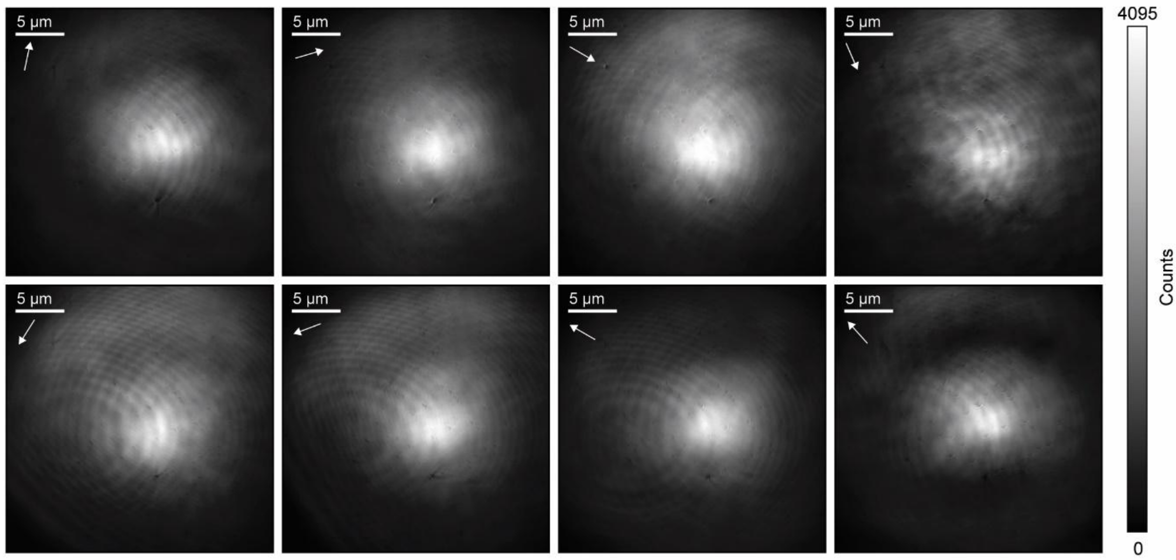


**Fig S1 Single-angle oblique illumination.** 40 nm gold sample illuminated from various directions by adjusting the beam position in the objective's back focal plane using dual-axis galvanometer mirrors. The illuminated region remains in a fixed position despite rotation from the galvanometer mirrors.

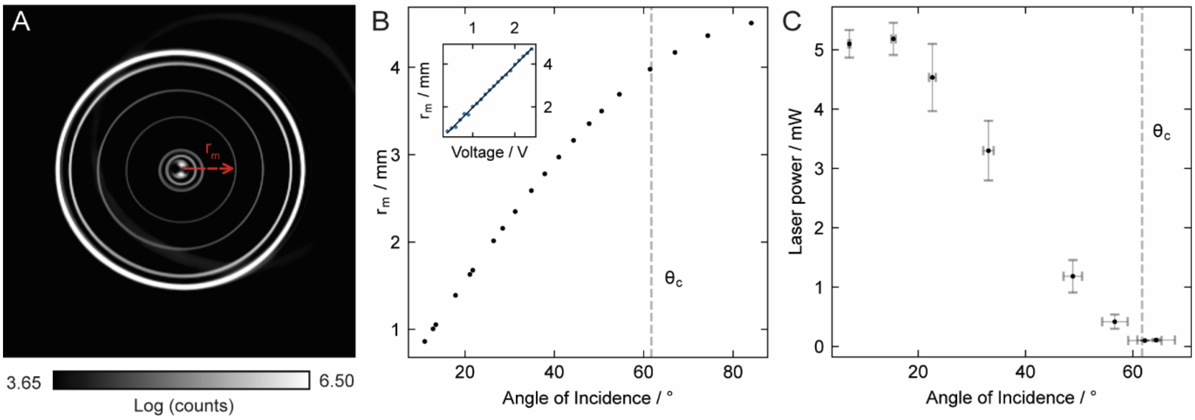


**Fig S2 Angle of incidence calculation and incident power.** (A) Reimaged reflected light at the back focal plane of the objective shown for six different incidence angles, superimposed on one another. (B) Radial measurements and corresponding calculated incidence angle. The inset shows the applied command voltage signal sent to the galvo mirrors plotted against the resulting radial measurement. (C) Illumination laser power used during measurements depicted in Fig. 3.

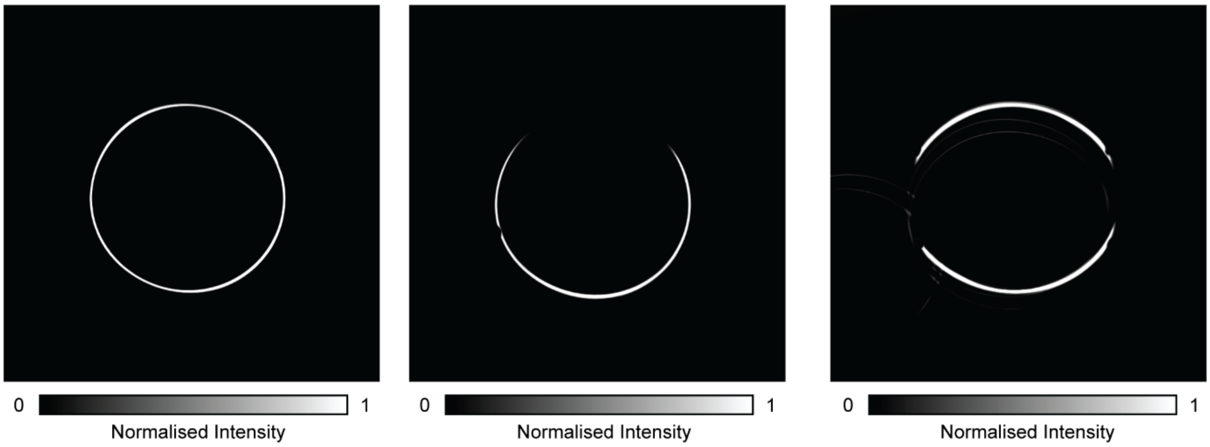


**Fig S3 Total internal reflection alignment channel.** Images from the total internal reflection alignment channel when scanning above the critical angle. Left: Optimal alignment. Middle: Off-centre alignment. Right: Non-circular scanning.

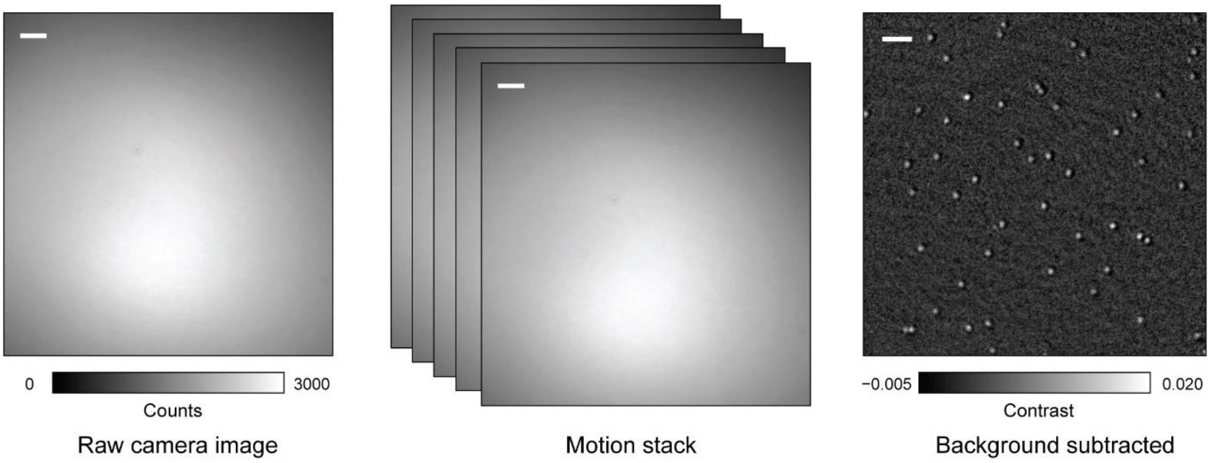


**Fig S4 Motion based flat fielding.** Background removal through motion-based flat fielding and image processing. A stack of 20 laterally translated raw images were acquired. Each raw image is then normalised by the median of the stack. A 2D median filter with a kernel size of 15 was then applied and subtracted from the original image, revealing the underlying 20 nm gold nanoparticles measured at 68° with ~0.04 kW/cm$^2$ power density. Scale bar: 1 µm.

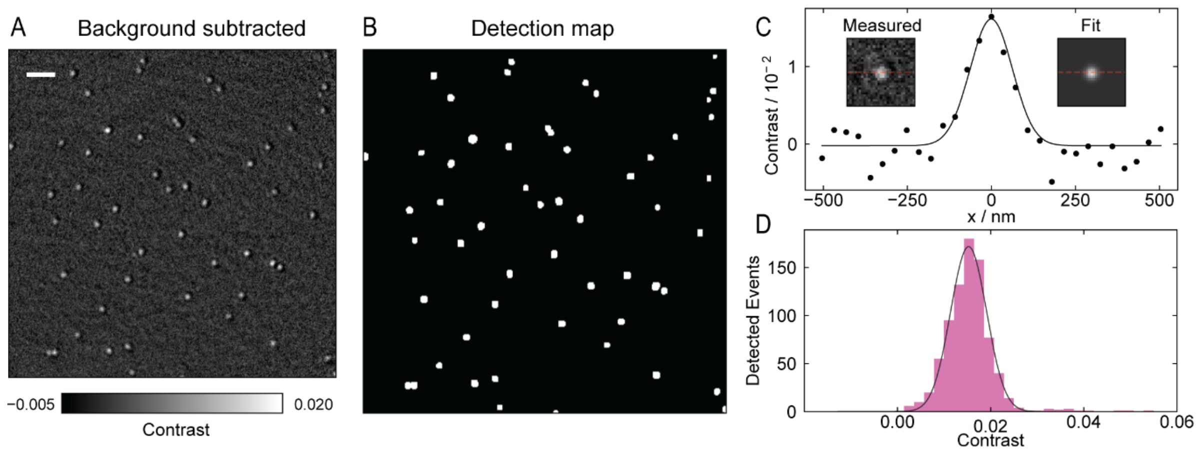


**Fig S5 Particle detection and fitting procedure.** (A) – Background subtracted image used for detection and fitting. (B) A detection map created by maxima/minima filtering followed by thresholding. (C) Detected regions of interest were then fitted with an experimental point spread function, and the peak magnitude extracted to estimate the GNP contrast. (D) Resulting distribution of particle contrasts. Scale bar: 1 µm.

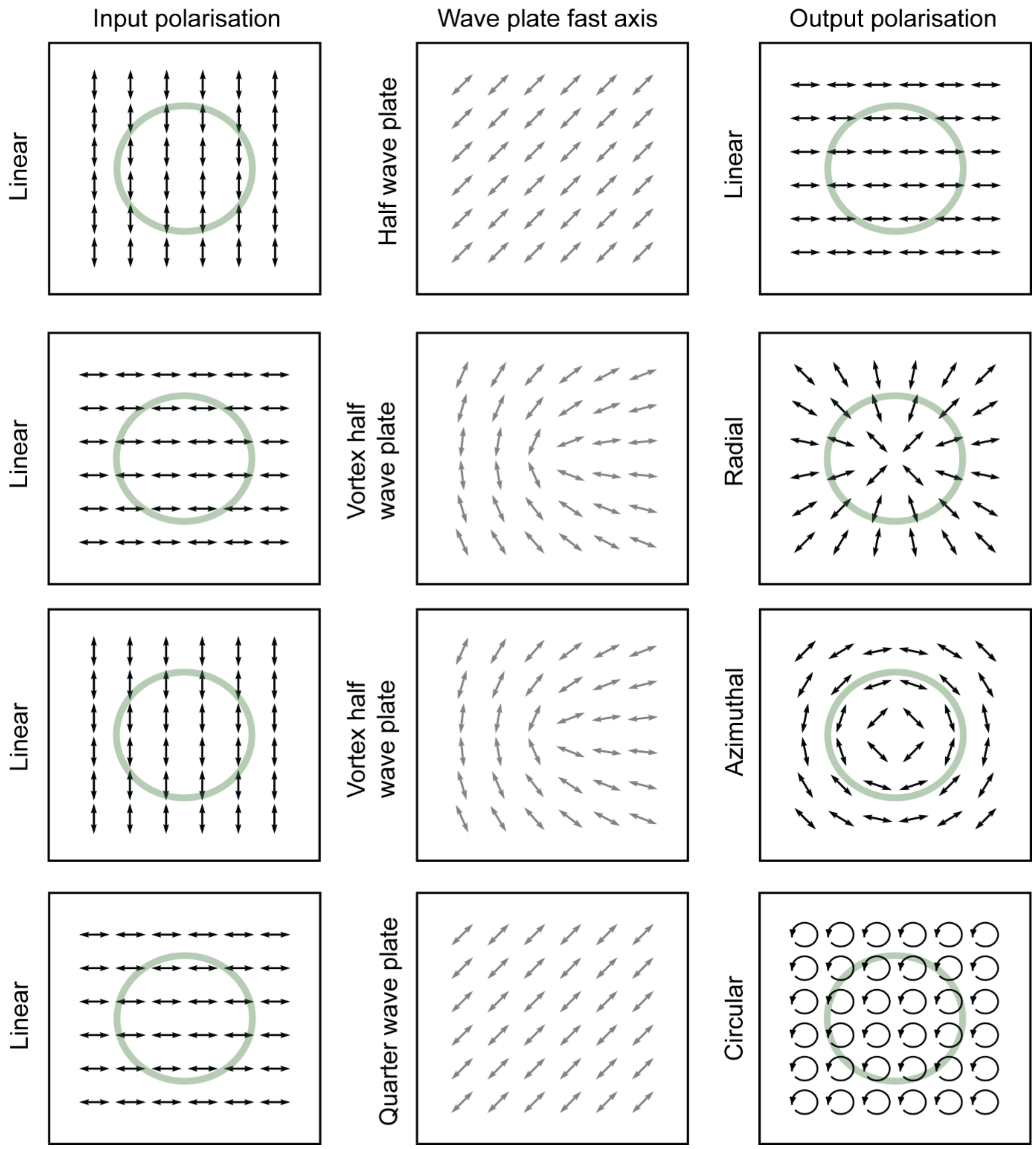


**Fig S6 Polarisation**. Illustration of various polarisation states achievable using different wave plates with linearly polarised incident light. For rotational scanning at high incidence angles, these configurations result in different illumination polarisations at the sample. High incidence, scanned, linearly polarised light produces a mixture of s- and p-polarised light, depending on the azimuthal angle during scanning. Radially polarised light results in predominantly p-polarised illumination. Azimuthally polarised light results in s-polarised illumination. Circularly polarised light results in a constant mixture of s- and p-polarised illumination for all azimuthal angles during scanning.

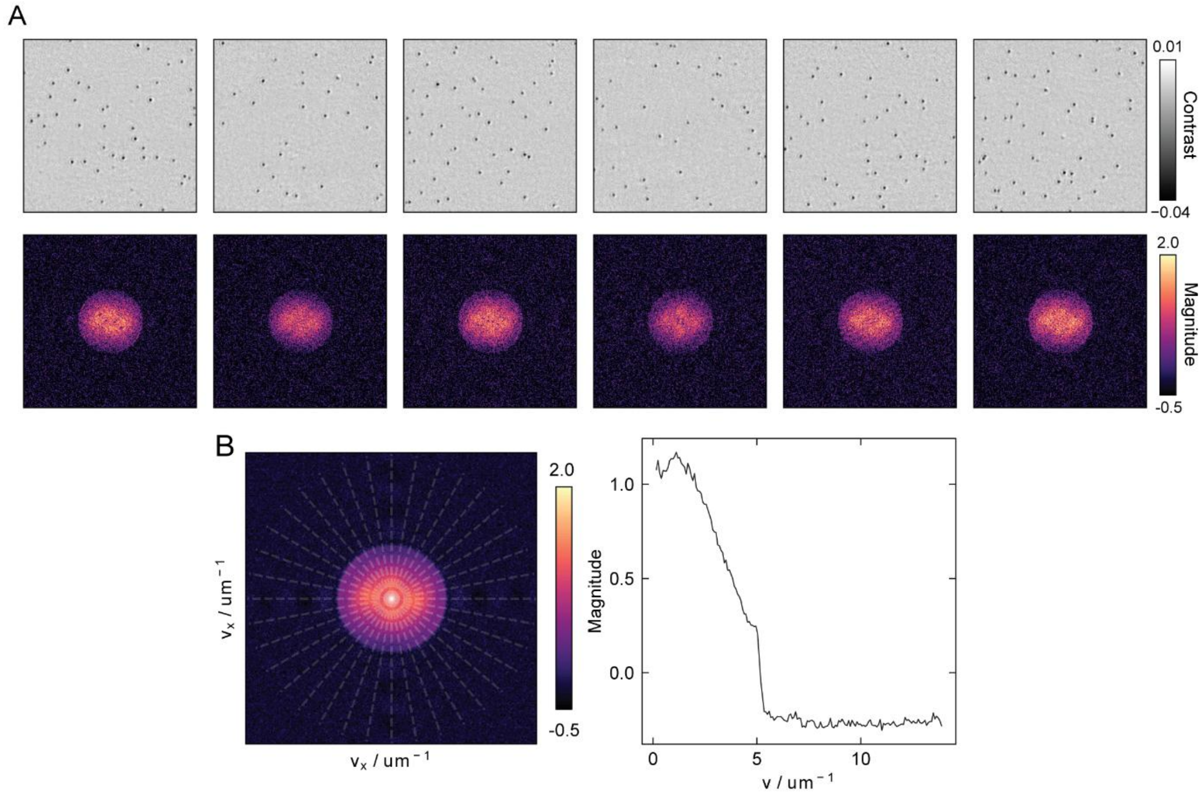


**Fig S7 Fourier spectra calculation for 20 nm GNPs.** (A) A 2D Fast Fourier Transform (FFT) algorithm processes images of the different sections of the sample. The resulting frequency spectra were shifted to centre the zero-frequency component. The magnitude spectrum was computed for each image and averaged to obtain the final Fourier spectrum (B). To derive the radial profile of the spectrum, 36 equally spaced line cuts were taken and averaged.

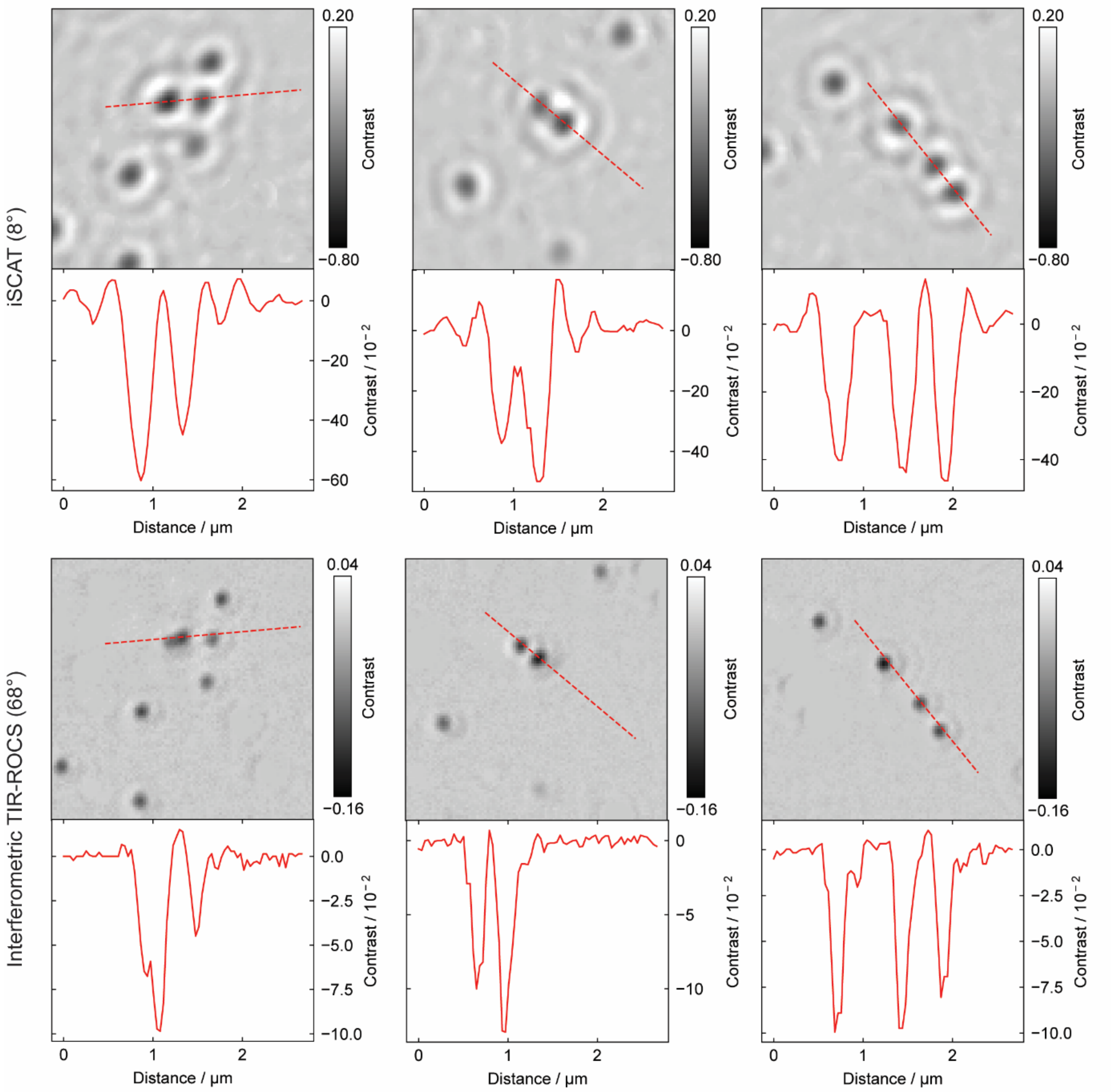


**Fig S8 Imaging of neighbouring 40 nm gold nanoparticles.** Top row: iSCAT (~8°); Bottom row: interferometric TIR-ROCS (~68°). The corresponding linecuts below the images illustrate the enhanced resolution achieved with TIR-ROCS.

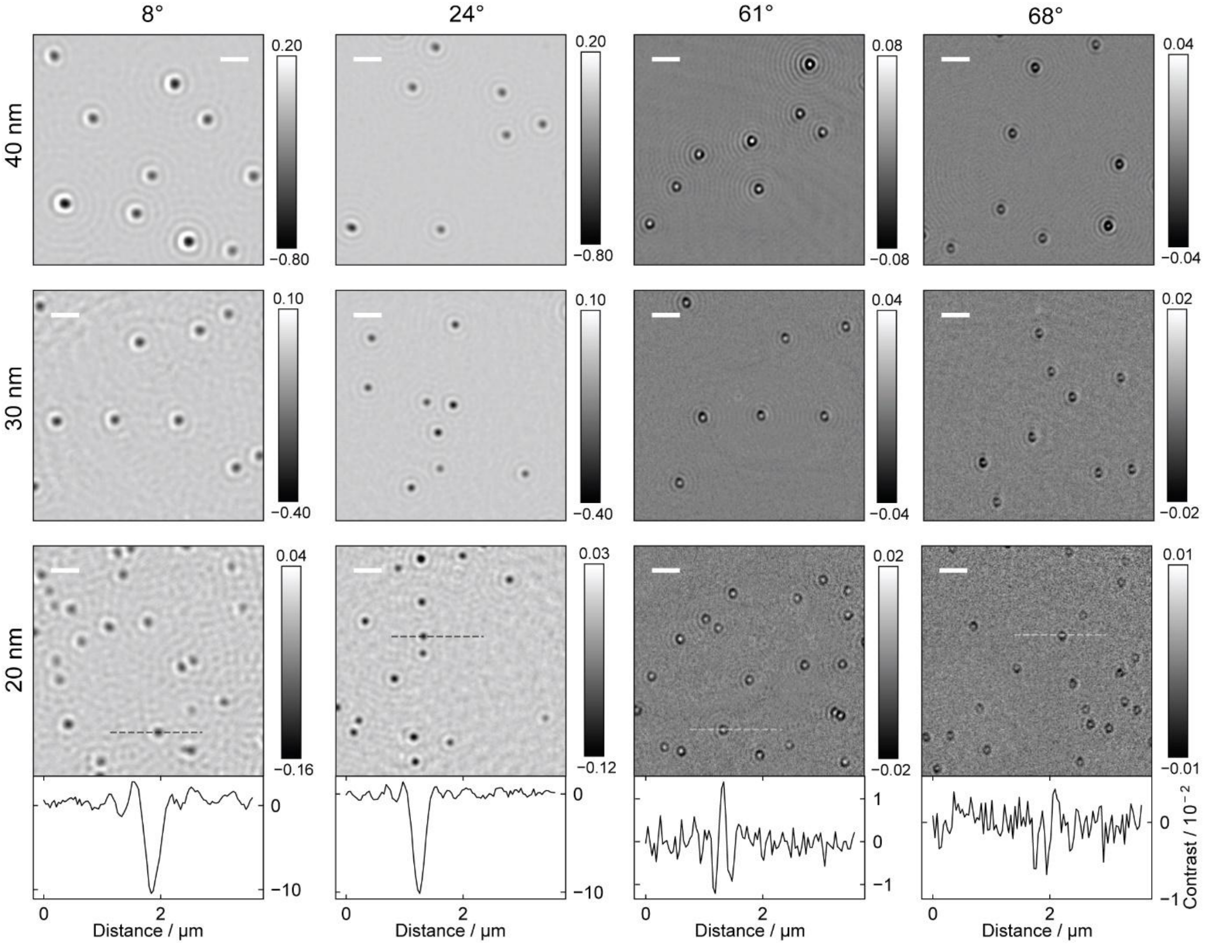


**Fig S9 Nanoparticle imaging as a function of incidence angle (radially polarised light)**. Images of gold nanoparticles (40, 30, 20 nm) measured using interferometric ROCS across various angles of incidences at 5 ms integration time. Predominantly p-polarised light illuminated the sample at high incidence angles, resulting from radially polarised light entering the objective. Accompanying line cuts are for 20 nm GNPs. Colour bars: Contrast. Scale bars: 1 µm.

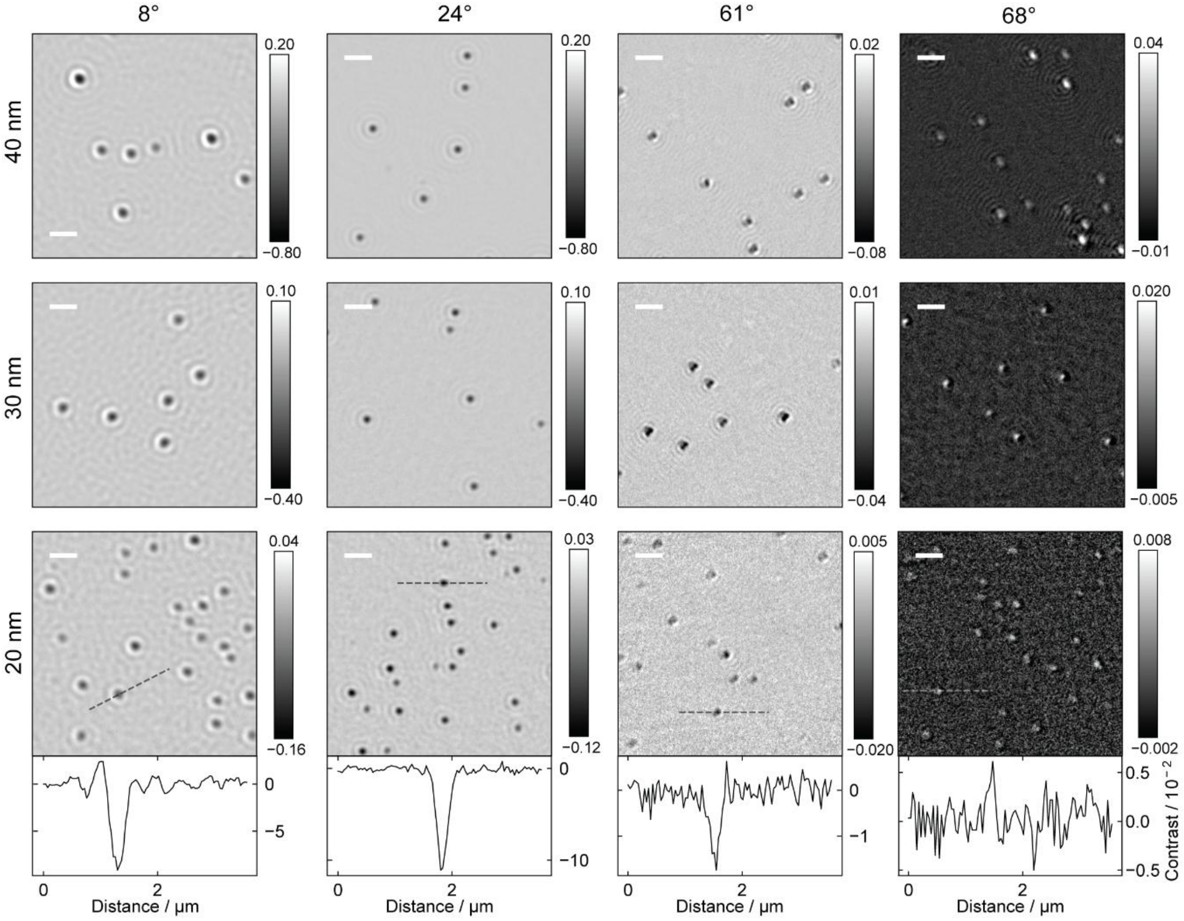


**Fig S10 Nanoparticle imaging as a function of incidence angle (circularly polarised light)**. Images of gold nanoparticles (40, 30, 20 nm) measured using interferometric ROCS across various angles of incidence at 5 ms integration time. A constant mixture of s- and p-polarised light illuminated the sample at all azimuthal angles, resulting from the circularly polarised light entering the objective. Accompanying line cuts are for 20 nm GNPs. Colour bars: Contrast. Scale bars: 1 µm.

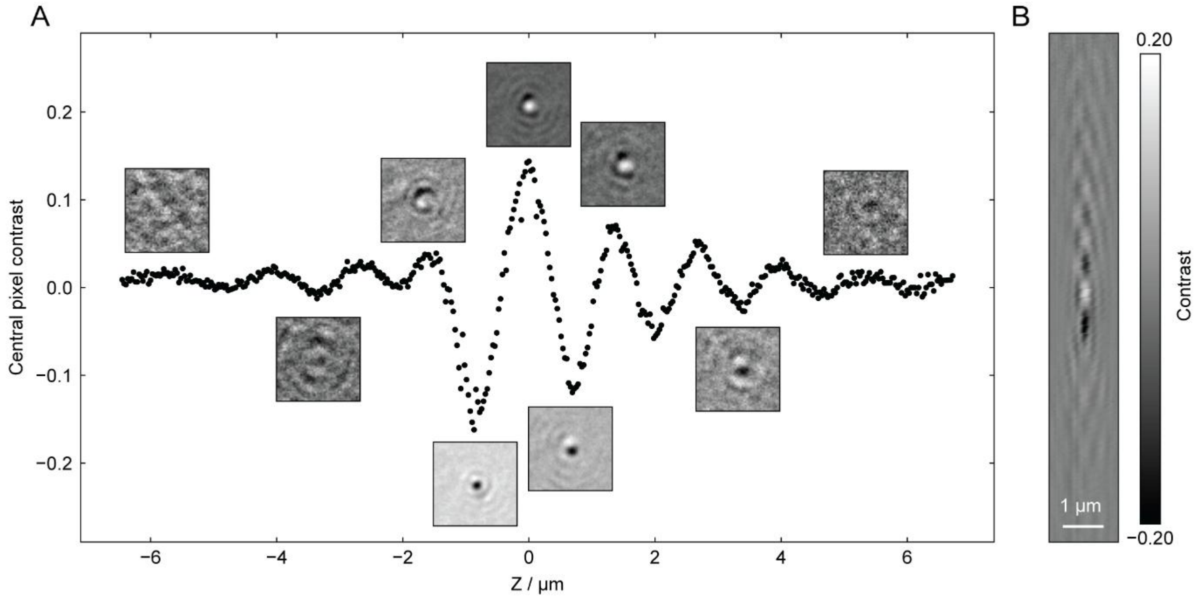


**Fig S11 Contrast variation with Z-position.** (A) Measured contrast of the central pixel of a 40 nm gold nanoparticle in TIR-ROCS (~68°) under s-polarised illumination, as the coverslip is translated through the objective focus in ~30 nm steps. High-contrast signals are observed at both constructive and destructive interference peaks, exhibiting similar contrast levels. (B) The measured YZ profile of the interferometric point spread function is also shown.

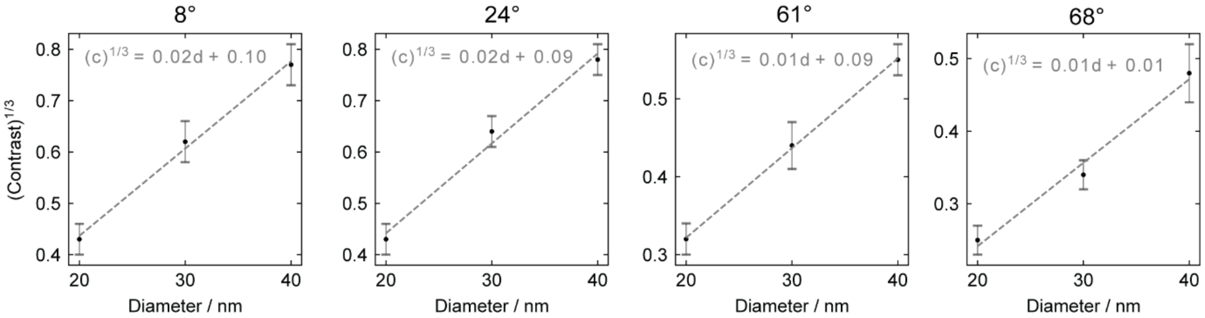


**Fig S12 Contrast scaling with particle size**. The diameter-cubed contrast scaling with particle size is confirmed using 40, 30, and 20 nm gold nanoparticles at increasing incidence angles. Analysis performed using data from Fig. 3.

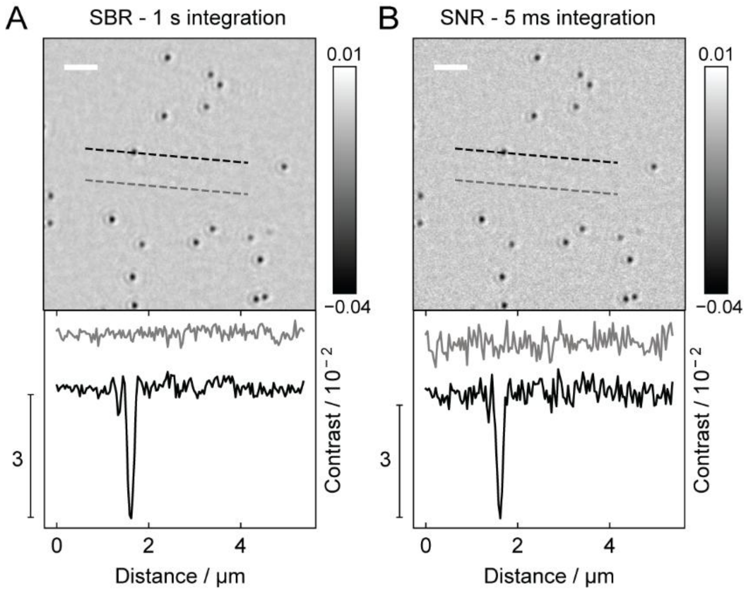


**Fig S13 Signal-to-background and signal-to-noise ratio calculations.** (A) Signal-to-background ratio (SBR) for 20 nm GNPs at 61°, calculated by comparing the standard deviation of the background line cut to the average particle contrast at 1 s integration time. (B) Signal-to-noise ratio (SNR) for the same GNPs, calculated at 5 ms integration, where there is significant shot noise contribution. Colour bars: Contrast. Scale bars: 1 µm.

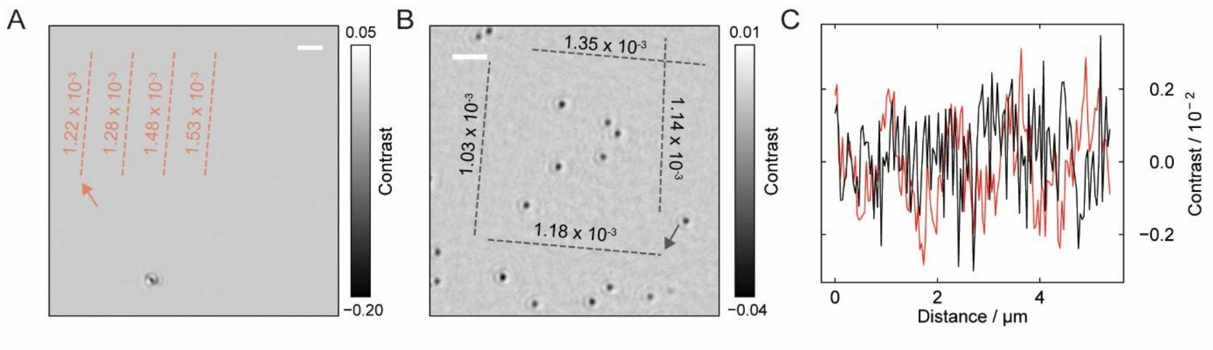


**Fig S14 Background comparison of sparse and dense samples.** (A) A sparse sample of 40 nm gold nanoparticles measured at 61°. The sampled line cuts are indicated by the dashed red line, with their corresponding standard deviations provided. The highlighted line cut is used for comparison in (C). (B) The same analysis is performed for a higher-density sample of 20 nm gold nanoparticles measured at 61°, with linecuts indicated by the dashed black line. (C) Comparison of the indicated line cuts from the sparse and high-density samples, showing similar background noise levels.
